# Immune complexes as culprits of immunopathology in severe COVID-19

**DOI:** 10.1007/s00430-022-00743-8

**Published:** 2022-07-23

**Authors:** Philipp Kolb, Sebastian Giese, Reinhard Edmund Voll, Hartmut Hengel, Valeria Falcone

**Affiliations:** 1 Faculty of Medicine, Institute of Virology, Freiburg University Medical Center, Albert-Ludwigs-University of Freiburg, Freiburg, Germany; 2 Faculty of Medicine, Department of Rheumatology and Clinical Immunology, Freiburg University Medical Center, Albert-Ludwigs-University of Freiburg, Freiburg, Germany

**Keywords:** COVID-19, Immunopathology, Immune complexes, Fc-gamma receptors

## Abstract

Infection with the pandemic human coronavirus SARS-CoV-2 elicits a respiratory tract disease, termed Coronavirus disease 2019 (COVID-19). While a variable degree of disease-associated symptoms may emerge, severe COVID-19 is commonly associated with respiratory complications such as acute respiratory distress syndrome (ARDS), the necessity for mechanical ventilation or even extracorporeal membrane oxygenation (ECMO). Amongst others, disease outcome depends on age and pre-existing conditions like cardiovascular diseases, metabolic disorders but also age and biological sex. Intriguingly, increasing experimental and clinical evidence suggests that an exacerbated inflammatory response and in particular IgG immune complexes (ICs), significantly contribute to severe and prolonged COVID-19 disease progression. Vast amounts of deposited, unresolved ICs in tissue are capable to initiate an exaggerated Fc gamma receptor (FcγR) mediated signalling cascade which eventually results in common IC-associated organ diseases such as vasculitis, glomerulonephritis and arthritis, comorbidities that have been frequently reported for COVID-19. Moreover and independent of deposited ICs, very recent work identified soluble ICs (sIC) to be also present in the circulation of a majority of severely ill patients, where their systemic abundance correlated with disease severity. Thus, detection of circulating sICs in patients represents a potential marker for critical COVID-19 disease progression. Their detection early after clinical deterioration might become an indicator for the requirement of prompt anti-inflammatory treatment. Here, we review the role of ICs in COVID-19 progression, their possible origins and potential intervention strategies.

**Graphical abstract:**

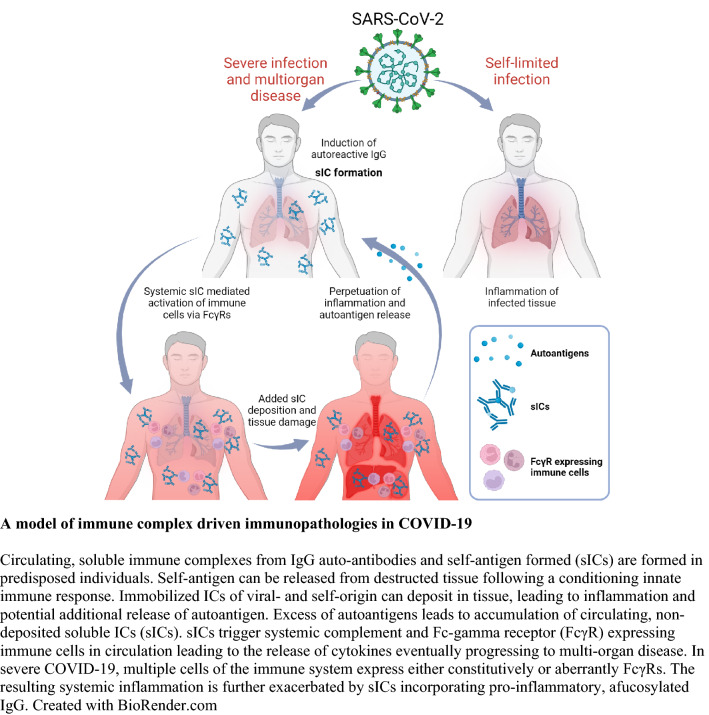

## Immune complexes and their effect on immune cells

Following the invasion of a pathogen, several arms of the human immune system become activated. Initially, cells of the innate immune system, such as dendritic cells (DC), macrophages (M), natural killer cells (NK) and granulocytes, together with the complement cascade, will cooperate to control pathogen replication and dissemination. Besides representing a potent first line of defence, the combined innate response is also a precondition to induce antigen-specific adaptive immune responses predominantly elicited by T (TC) and B cells (BC) roughly 5–14 days after symptom onset. Among others, the adaptive response brings forth pathogen-specific antibodies of the IgG subclass. Naturally, IgG will bind to its respective antigen, forming so-called immune complexes (ICs). IgG-ICs can be transiently found deposited in tissue (ICs) or soluble (sICs) in the human circulation. Both, circulating as well as deposited IgG-ICs will usually be rapidly cleared by phagocytes, which, to this end, express specific receptors recognizing the Fc part of an IgG molecule (Fcγ bound by FcγRs). Clearance of IgG-ICs is primarily mediated via FcγRIIA and FcγRI, expressed by granulocytes such as neutrophils and monocyte-derived cells like dendritic cells or macrophages. In addition to eliminating ICs, FcγRs are also crucial signal transducers of immune cells, whereby activating (e.g. FcγRIII/CD16) and inhibitory FcγRs (e.g. FcγRIIB/CD32B) are constitutively expressed in a cell-type-specific manner [1]. sIC interaction with FcγRs is governed by the IgG glycan profile [2], with activating FcγRs showing enhanced affinity to desialylated and afucosylated IgG. Further, FcγRs are sensitive to the molecular size of sICs with multimeric, large sICs showing stronger reactivity compared to smaller sICs [3, 4]. However, if IgG-ICs cannot be cleared and remain in circulation, they can cause severe systemic and long-lasting inflammation. These complexes are referred to as circulating ICs (CIC) as a characteristic of certain autoimmune diseases. More generally, soluble ICs (sICs) describe all non-deposited IgG complexes in clinical as well as experimental settings. An autoimmune disease where sICs can drive systemic inflammation is systemic lupus erythematosus (SLE). SLE is characterized by circulating autoreactive antibodies that deposit in tissues, including skin, kidneys, and brain. Here, IC deposition elicits a progressive inflammatory response resulting in tissue damage [5] and further release of autoantigens at a given point [6–9]. This self-propelling process eventually drives severe systemic inflammation, which can often only be controlled by the administration high-dose glucocorticoids and cyclophosphamide. In very severe cases, these drugs have been combined with the systemic removal of pro-inflammatory factors from the circulation via plasmapheresis [10]. While IC-mediated local inflammation is commonly associated with complement activation and subsequent immune cell recruitment and stimulation, FcγR activation by sICs likely plays an even more important role as the club of immune cells expressing FcγRs is continuously growing. Next to monocytes, granulocytes, platelets and NK cells, recent studies showed that subsets of γδ-T cells can also express an FcγR, specifically FcγRIIIA [11]. A recent study using a panel of reporter cells expressing individual FcγRs revealed that not only the inhibitory FcγRIIB but especially the activating FcγRs IIA (genotype H) and FcγRIIIA showed strong reactivity towards sICs, albeit the profile of effector responses elicited in primary NK cells differed between sICs and immobilized ICs. Recently, αβ-T cells were shown to aberrantly express high levels of FcγRIIIA in severely diseased COVID-19 patients where they exert polyclonal TCR-independent highly cytotoxic T cell responses [12].

## Immune complex-mediated inflammation in viral infections

IC-mediated repercussions evoked by viral infections are collectively termed antibody-dependent enhancement (ADE). ADE can be broadly categorized into two different molecular mechanisms. First, ADE can refer to antibody-dependent enhanced infection. Here, IgG-opsonized virions show enhanced virus uptake and replication in FcγR expressing cells such as macrophages. The best-documented example in this regard is Dengue virus infection, where there is a direct correlation between in vitro ADE and clinical manifestation [13, 14]. Likewise, there is in vitro evidence for enhanced infection of FcγR expressing macrophages via ADE using SARS-CoV-1 and MERS [15–17]. Recently, Junqueira et al. were able to show that a fraction of monocytes found in patients is infected by SARS-CoV-2 and that this infection is mediated by FcγRs and virus-specific IgG. However, although infection resulted in initial viral gene expression, it was ultimately found to be abortive [18]. While in this publication it was shown that infected monocytes could contribute to systemic inflammation via inflammasome activation and subsequent pyroptosis, there is still no evidence supporting ADE to play a dominant role in COVID-19 disease progression. In line with this finding, others also argue against macrophages as productive host cells of SARS-CoV-2 infection [19]. Second, ADE can also refer to enhanced immune cell activation by activating FcγRs and subsequent inflammation. Here, similar to autoimmune diseases like SLE, uncleared IC deposition can lead to severe clinical manifestations [20] such as glomerulonephritis [21–23], vasculitis [24] or bone erosion [25]. Antibodies associated with this form of ADE are typically non-neutralizing and associated with immunopathology, predominantly in respiratory viral infections such as RSV and measles [26–28]. Deducing from this, pro-inflammatory ADE likely plays an important role in COVID-19 disease progression as well. Specifically, there is mounting evidence that complexed IgG is a key ingredient for exacerbated disease. Further, severe COVID-19 often shows striking similarities to autoimmune diseases like SLE or rheumatoid arthritis, both of which are characterized by the emergence of autoantibody-derived sICs.

## Potential origins of ICs in COVID-19

Autoantibodies can potentially be generated during SARS-CoV-2 infection in two ways. Next to molecular mimicry, a phenomenon where viral antigens are activating immune responses towards autoantigens [29], the inflammatory host response following a SARS-CoV-2 infection could induce autoantibody formation in predisposed patients. While clinical evidence for molecular mimicry is still missing, the latter mechanism has been frequently described in COVID-19. Amongst others, an unexpectedly high percentage of COVID‐19 patients, clinically suspected to have heparin-induced thrombocytopenia, developed high titers of anti‐platelet-factor-4(PF4)/heparin antibodies [30]. Along these lines, vaccine-induced thrombotic thrombocytopenia (VITT) following the administration of SARS-CoV-2 vector-based vaccines has also been linked to anti-PF4 autoantibody induction [31]. In addition, there have been numerous reports on elevated levels of autoantibodies associated with rheumatological diseases in COVID-19 patients but also against immunomodulatory proteins including interferons, cytokines, chemokines, complement components and cell-surface proteins [32–36]. In particular, the presence of anti-phospholipid antibodies has been linked to thromboembolic events [37, 38]. In certain cases, autoantibodies were induced following infection [36, 39]. To conclude, pro-inflammatory ADE in COVID-19 could be a consequence of sIC formation following autoantibody induction. While it cannot be excluded that viral antigens are part of sIC formation, as immune complex vasculitis has been observed following mRNA vaccination (BNT162b2) [40] and shed S-antigen was detected following mRNA vaccination (mRNA-1273) [41] as well as in plasma of patients with severe disease [42], there is no conclusive evidence on persisting, circulating SARS-CoV-2 antigens during later stages of severe COVID-19 when viral loads are vanishing. However, continuous antigen production is required for sustained sIC formation. In line with this, a recent study proved the presence of serum-derived sICs in severe COVID-19, which were devoid of SARS-CoV-2 antigen [43]. Although not directly identifying autoantibodies as culprits, the authors show that sICs are not formed following administration of either heterologous (Vaxzevria/Spikevax) or homologous (Comirnaty) prime-boost vaccination, demonstrating that spike-antigen expression per se does not induce sIC formation. Another piece to the puzzle is also provided in this study, as in a sizeable group of patients SARS-CoV-2 specific IgG is detected only after the emergence of sICs.

## IC-mediated immune cell activation and systemic inflammation in COVID-19

While there are more hypothetical considerations on how soluble immune complexes would act systemically in COVID-19 [44], several studies directly link deposited immune complexes and FcγR activation to tissue damage in COVID-19. One study showed that FcγRIIA-mediated activation of platelets is linked to thrombocytopenia in critically ill patients [45, 46]. Another study reported that enhanced eosinophil-mediated inflammation in the respiratory tract of critically ill and deceased COVID-19 patients is associated with FcγR signaling in myeloid cells [47]. Finally, neutrophil activation by immune complexes via FcγRIIA was suggested to negatively impact COVID-19 progression [48]. Here, the authors demonstrate that ICs resulted in a more inflammatory neutrophil activation profile when containing anti-SARS-CoV-2 IgG from severely diseased patients compared to IgG from mildly diseased patients. As FcγRs are sensitive to IgG glycan modifications, this is in line with other studies showing that the increase in afucosylated IgG correlates with COVID-19 severity [49–51]. Further, afucosylated anti-SARS-CoV-2 IgG occurring in COVID-19 was shown to be directly linked to FcγRIIIA-mediated activation irrespective of disease severity [43, 49] and was associated with alveolar macrophage-driven inflammation [52]. This finding is reminiscent of SLE, where de-sialylated and afucosylated IgG may exert stronger pro-inflammatory effects [2, 53]. To make matters worse, as mentioned above, a previously unknown population of αβ-TC expressing high levels of FcγRIIIA and possessing increased cytotoxic functions has been described to emerge during severe COVID-19 [12]. In light of the many reports showing autoantibody formation in COVID-19 and the recent discovery of sICs being present in patient circulation [43], it is highly likely that severe COVID-19 follows a similar, if not even higher, combined local IC and systemic sIC driven immunopathology as observed in SLE where both ligands lead to excessive FcγRIIIA/CD16 activation. Fittingly, the same study that identified sICs in the circulation of severely and critically diseased patients also showed that sIC reactivity significantly correlated with the severity of disease [43]. The authors further demonstrated that sIC reactivity was comparable to and even slightly exceeded that of patients with active SLE. Naturally, such a systemic event might also be an explanation for the generally observed systemic increase in pro-inflammatory cytokines during COVID-19 [54]. To conclude, it can be speculated that the large arsenal of highly reactive FcγRIIIA/CD16 + immune cells, including TC in severe COVID-19, is excessively activated by sICs, which leads to severe systemic inflammation and multi-organ disease. One may even surmise that COVID-19-associated Multisystem Inflammatory Syndrome in children or adults (MIS-C/A) [55–58] is also connected to sIC formation and FcγR activation. Only recently a comprehensive multi-omics study of a large multi-institutional paediatric MIS-C cohort analysed multiple soluble biomarkers and applied proteomics as well as single-cell gene expression approaches [59]. This investigation revealed some similarities between severe COVID-19 and MIS-C such as the emergence of auto-antibodies, but also remarkable differences such as the amounts of spike antigen in the circulation and genetic markers such as HLA class I alleles [59]. Accordingly, the two SARS-CoV-2 caused diseases appear to be distinct with separate immunopathologies, but this insight should not obviate the need to search for the presence of sICs in MIS-C.

## Therapeutic implications and future research needs

In recent months it has become obvious that COVID-19 can bear some striking resemblance with autoimmune diseases such as SLE. The emergence of an autoantibody response against a multitude of unrelated self-antigens, enhanced afucosylated IgG and IC-mediated clinical manifestations such as vasculitis or glomerulonephritis make this syndrome unique among the known respiratory infections. This became even more obvious when highly reactive sICs, a hallmark of active SLE, were detected in the circulation of severely diseased COVID-19 patients, but not in the blood of patients with acute respiratory distress syndrome (ARDS) in response to other etiologies [43]. The remaining question is whether we can utilize this knowledge to optimize current intervention or relief strategies. Due to the systemic inflammation often observed in critically diseased patients, it is not surprising that one of the most successful immediate treatment strategies is the administration of corticosteroids. Complement inhibition in COVID-19 also showed promising effects by reducing inflammation and preventing lung injury [60, 61]. Alternatively, monoclonal antibodies targeting SARS-CoV-2 showed promising efficacy, although there is a widely observed escape by the most recent Omicron variants [62]. Considering the impact of IgG complexes on systemic inflammation, it is, however, important to exclude any adverse effect in this direction when administering IgG preparations such as SARS-CoV-2 specific monoclonal antibodies, antibody cocktails or reconvalecent-immunoglobulin administered as prophylactic intervention against the progression of SARS-CoV-2 infection to severe COVID-19. If ICs might be formed following IgG administration they could bear the risk of exacerbating disease via enhanced complement and FcγR activation. An absence of such side effects would support the hypothesis that sICs actually contain autoantibodies and self-antigen when they occur in severely ill COVID-19 patients. The removal of sICs could be a promising strategy to alleviate severe disease. Indeed, recent studies published promising results regarding the usefulness of plasmapheresis [63, 64], which used to be a last resort effort to treat highly active SLE. This procedure is able to remove sICs, autoreactive IgG and cytokines altogether. Still, due to the costly and laborious process, it will likely remain the last resort effort to treat the most severe cases of immunopathology in COVID-19. Lastly, a potential strategy to block the FcγR driven part of IC-mediated immunopathology could simply be the administration of large amounts of non-specific human IgG (IVIg). In absence of a specific antigen, this should not lead to the formation of sICs. Indeed, there is evidence that the administration of IVIg can improve the clinical outcome and significantly reduce mortality in COVID-19 65,66. While it is still unclear if we identified all major culprits of immunopathology in COVID-19, recent work lifted the curtains to reveal immune complexes as a unique but also familiar facet of this novel disease.
